# Fibre-based spectral ratio endomicroscopy for contrast enhancement of bacterial imaging and pulmonary autofluorescence

**DOI:** 10.1364/BOE.10.001856

**Published:** 2019-03-15

**Authors:** Helen E. Parker, James M. Stone, Adam D. L. Marshall, Tushar R. Choudhary, Robert R. Thomson, Kevin Dhaliwal, Michael G. Tanner

**Affiliations:** 1 EPSRC Proteus IRC Hub in Optical Molecular Sensing & Imaging, Centre for Inflammation Research, Queen's Medical Research Institute, University of Edinburgh, Edinburgh, UK; 2 Centre for Photonics and Photonic Materials, Department of Physics, University of Bath, Bath, UK; 3 Institute of Biological Chemistry, Biophysics and Bioengineering, School of Engineering and Physical Sciences, Heriot-Watt University, Edinburgh, UK; 4 The Roslin Institute and Royal (Dick) School of Veterinary Studies, University of Edinburgh, Edinburgh, UK; 5 Scottish Universities Physics Alliance (SUPA), Institute of Photonics and Quantum Sciences, School of Engineering and Physical Sciences, Heriot-Watt University, Edinburgh, UK

## Abstract

Fibre-based optical endomicroscopy (OEM) permits high resolution fluorescence microscopy in endoscopically accessible tissues. Fibred OEM has the potential to visualise pathologies targeted with fluorescent imaging probes and provide an *in vivo in situ* molecular pathology platform to augment disease understanding, diagnosis and stratification. Here we present an inexpensive widefield ratiometric fibred OEM system capable of enhancing the contrast between similar spectra of pathologically relevant fluorescent signals without the burden of complex spectral unmixing. As an exemplar, we demonstrate the potential of the platform to detect fluorescently labelled Gram-negative bacteria in the challenging environment of highly autofluorescent lung tissue in whole *ex vivo* human lungs.

## 1. Introduction

Fluorescence microscopy has demonstrated itself as a powerful tool for visualising biological pathways. Diverse processes of living cells can be directly visualised in many biological contexts across *in vitro* and *in vivo* settings, enabled by a plethora of molecular imaging probes [1,2]. This is due to the high sensitivity and specificity of fluorescence microscopy, particularly when endogenous and exogenous fluorescent signatures are utilised together effectively [3]. Translating high resolution molecular microscopy utilising fibred OEM to human disease has the potential to deliver unprecedented molecular insights, particularly if coupled with targeted molecular imaging probes.

Fibred OEM is enabled by small diameter flexible fibre bundles that permit microscopic imaging of organ systems such as the gastrointestinal tract [4,5], the urinary tract [6,7], and the respiratory tract [8,9], which is the focus of this paper. Important pathogenic or biological information contained in fibred OEM is increased by increasing the number of colour channels [9], alongside highlighting disease processes with multiple targeted fluorescent probes (SmartProbes). These SmartProbes span a wide breadth of utility within human lung tissue imaging, from direct imaging of bacterial burdens [10] to imaging of molecules, such as matrix metalloprotease [11], lysyl oxidases [12], neutrophil elastase [13], and thrombin [14], that are known to be significantly elevated in pulmonary diseases such as adult respiratory distress syndrome, pulmonary fibrosis and lung cancer [15–18].

Unsurprisingly, there are major limitations of single colour (wavelength) imaging which are also exemplified by the increasing recognition of multiplexing immunohistochemical platforms in modern pathology. In this regard, many permutations of fluorescence imaging systems can be realised for multicolour imaging, each with its own associated advantages and disadvantages. A typical method of achieving multicolour fluorescence imaging is through the use of various illumination wavelengths and carefully chosen filter sets. Images are usually best captured on a monochromatic camera due to its comparatively higher quantum efficiency than a colour camera. Each colour channel then contains only intensity information within the spectral band defined by the filter set and images are subsequently false-coloured [8,9], meaning that fine spectral detail within the band is entirely lost. Given that many fluorophores have wideband emission spectra, it is exactly this spectral detail that can be taken advantage of to unmix the fluorescent signals representing crucial biological information within the imaging field of view (FOV). For example, collagen and elastin abundantly present in lung tissue enable label free *in vivo* tissue pattern delineation [19], but can complicate the detection of fluorescent probes if there are significant spectral overlaps. Recently, we described the development and clinical translation of a targeted SmartProbe labelling Gram-negative bacteria using an environmentally sensitive fluorophore, nitrobenzoxadiazole (NBD) that resides in the green region of the optical spectrum [20]. Its fluorescent signal is dependent on the polarity of its environment and hence the probe is silent (non-fluorescent) until bacterial membrane insertion – a requirement for clinical imaging where wash steps cannot be performed. The probe was shown to have Gram selectivity across a broad and clinically relevant panel of organisms, and was shown to not label mammalian cells. The Gram-negative specific probe was coupled with a commercially available OEM system. The motivation for using this wavelength of fluorophore was driven by the availability of clinically approved fibred OEM systems that could detect the SmartProbe in human disease. However, these single colour clinically available fibred OEM systems have technical limitations that preclude the potential to disentangle the strong lung autofluorescent signal which has a broad peak in the green region. Thus in these scenarios, spectral sorting of autofluorescence and SmartProbes can be advantageous.

Multispectral and hyperspectral imaging techniques exist to improve signal to noise ratio and identification of different spectral signals, particularly in environments where tissue autofluorescence is high and several fluorescent probes are in use simultaneously. There are many multi/hyperspectral imaging techniques afforded to microscopy, described elsewhere [21,22]. These techniques typically rely on dispersive optical elements, long acquisition times, and subsequent unmixing of spatially and spectrally dense data. However, in many imaging contexts there is *a priori* knowledge of potential spectral contributions. Thus extraction of useful information can be achieved with a significantly smaller data set than required for blinded spectral imaging. As such, to successfully resolve SmartProbes from tissue autofluorescence or to resolve spectrally similar SmartProbes from each other, acquiring dense spectral information may be redundant.

Thus motivated by the need to separate fluorescent targets during *in vivo* lung imaging contexts, we describe a simple inexpensive widefield imaging system, built from off-the-shelf optical components and coupled with a novel low-cost single use disposable imaging fibre bundle [23] which has been packaged to be readily introduced into endoscopes [24]. The imaging system utilises a single colour LED illumination source (470 nm), yet exploits ratiometric methods to enhance the contrast between similar fluorescent sources. This is achieved through calculating a spectral ratio value according to the relative proportion of light transmitted above and below a cut-off wavelength. We define the spectral ratio value as the short channel (approx. 470 nm – 605 nm) relative to the long channel (approx. 605 nm – 800 nm). We make use of the discrete fibre structure to perform ratiometric analysis on a core-by-core basis. Other methods of multispectral or hyperspectral imaging that are capable of resolving multiple wavelength sources [25–27], would not work without modification in this context due to complex mode patterns down the fibre cores. The guided mode patterns present in the two wavelength bands are not identical. As such it is vital to extract the intensity per fibre core per wavelength channel for computational processing and image reconstruction, rather than direct analysis of overlaid camera images from each band. This approach also removes the intrinsic pattern of the fibre cores from the resulting images.

Although the platform described here is single-colour illuminated, several illumination sources and spectral bands can be easily and inexpensively incorporated or adapted into the architecture to suit a variety of purposes, within the limits of target fluorescence intensity. We achieve two spectral bands through use of an optical chopper, with each channel overlaid on the camera multiplexed in time. A similar result could be achieved without a chopper but with the addition of a second camera. Side by side tiling of the two spectral bands on a single camera was considered. However, it was found that reducing the magnification of each channel to suit our detector size resulted in a pixel to core ratio that was prohibitively low. The platform adds significant versatility to low-cost widefield fibred OEM platforms that we have recently described [8,9]. As an initial proof-of-concept, we demonstrated the potential of the system to detecting bacteria pre-labelled with SmartProbe (NBD-PMX) in lung tissue. This has been the subject of significant efforts in signal processing and image analyses [28] as well as other modalities such as fluorescence lifetime imaging [29]. Our new approach offers a simple, readily translatable and effective solution to the technical challenge.

## 2. Methodology

This section describes the optical setup capable of acquiring images for ratiometric analysis (§ 2.1), the methodology of the image processing itself (§ 2.2), and the experimental methods used to demonstrate the capabilities of the imaging system (§ 2.3).

### 2.1 Optical setup

**Fig. 1:**
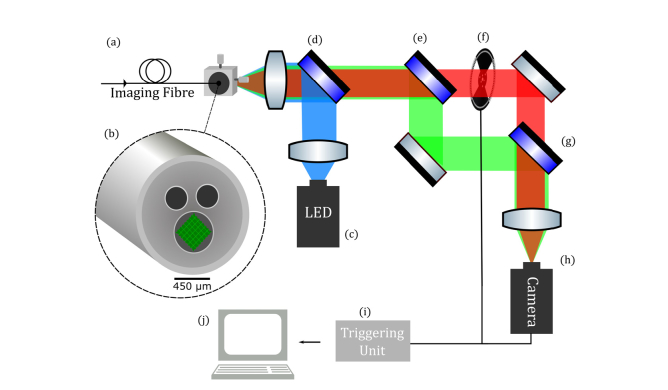
Ratiometric imaging system. Panoptes imaging fibre (a) consisting of a tessellated array of 8100 cores (b). A blue LED (c) is coupled via a dichroic mirror (d) into the fibre bundle. The fluorescence emission is separated by a second dichroic mirror (e) about 605 nm. The long wavelength path is interrupted by an optical chopper (f) and recombined with another dichroic mirror (g) onto a monochromatic camera (h). A PC (j) is used to control a triggering unit (i) with outputs to the camera and the chopper.

Our widefield fibred imaging platform is enabled by a novel multifunctional endoscopic fibre, Panoptes, developed within our group [23,24]. Panoptes is a low index contrast imaging fibre consisting of 8100 cores with a 450 µm corner to corner field of view, packaged alongside two capillary channels for the delivery and extraction of fluids, such as SmartProbes, see Fig. 1 (b)

. The rest of the hardware for our ratiometric imaging system consists entirely of off-the-shelf components and is depicted in Fig. 1.

A 470 nm LED (M470L3, Thorlabs) illumination source was collimated using an anti-reflection coated achromatic condenser lens (ACL2520-A, Thorlabs) and directed through a standard epi-fluorescence arrangement of excitation filter and dichroic (FITC-Ex01-Clin-25, Semrock). The excitation light was then coupled into our fibre bundle and mounted in an x-y translation stage (ST1XY-D, Thorlabs) through a microscope objective (10 × , 0.3 NA, UPLFLN 10X2, Olympus).

The emitted fluorescence from the target propagated through the fibre and, after passing through the epi-fluorescence dichroic and emission filter, was split into two optical paths according to a cut-off wavelength defined by the dichroic mirror (DMLP605R, Thorlabs). Into the long wavelength path, we placed an optical chopper (MC2000B, Thorlabs). The two paths were recombined using another dichroic mirror (DMLP605R, Thorlabs) and focused onto a monochromatic CMOS camera (GS3-U3-23S6M-C Grasshopper, Point Grey). A PC operated a triggering unit to synchronise the chopper rotation with camera acquisition. This ensured that the sequential full wavelength and short wavelength images were taken at 50 ms exposure time. From this, we easily derived a short wavelength channel (< 605 nm) and a long wavelength channel (> 605 nm) whilst acquiring images at 10 fps video rate.

The dichroic mirrors, shown in Fig. 1 (e) and (g), define the cut-off and hence a value that we refer to as the spectral ratio. The spectral ratio, R, is the ratio of normalised core intensity in the short channel, $I_{sc}$, divided by the normalised core intensity in the long channel, $I_{lc}$, and is given by,

$$R=\;\frac{I_{sc}}{I_{lc}}=\frac{\left[\sum_{i=1}^{n}p_{i}\left(m_{sct}\right)\right]{\left[\sum_{i=1}^{n}p_{i}(m_{scb})\right]}^{-1}}{\left[\sum_{i=1}^{n}p_{i}\left(m_{lct}\right)\right]{\left[\sum_{i=1}^{n}p_{i}(m_{lcb})\right]}^{-1}}$$ (1)

Where $m_{sct}$ and $m_{lct}$ are the $m^{th}$ cores in the short channel target image and long channel target image respectively. $m_{scb}$ and $m_{lcb}$ are the $m^{th}$ cores in the short channel brightfield image and long channel brightfield image respectively. $p_{i}$ is the $i^{th}$ pixel intensity of the $m^{th}$ core, n is the number of pixels belonging to the core.

**Fig. 2:**
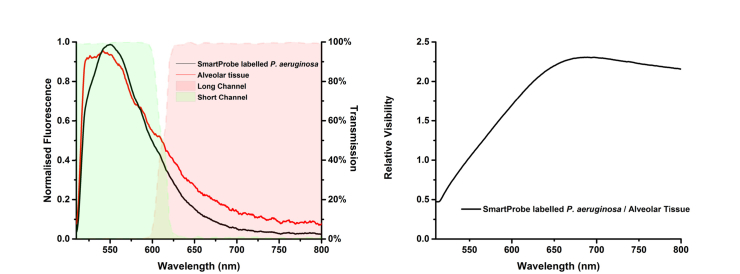
(left) Example fluorescence emission of SmartProbe labelled *P. aeruginosa* (black) and human lung (red) at excitation of 470 nm at 5.5 µW acquired using a spectrometer (USB2000 + VIS-NIR-ES, Ocean Optics) coupled into a spectroscopy setup analogous to our imaging arrangement. SmartProbe labelled bacteria have greater short wavelength contributions than typical lung tissue autofluorescence. Profile of dichroic mirrors used to separate the short and long channels determines the short channel wavelength range (green fill) and the long channel wavelength range (red fill). (right) Relative visibility of SmartProbe labelled bacteria defined as the relative spectral ratio value of SmartProbe labelled bacteria to lung tissue for a range of potential cut-off wavelengths.

Rather obviously, the optimum cut-off wavelength is dependent on the expected fluorescence of the targets to be imaged. In our case, the cut-off wavelength was selected to maximise the relative visibility of labelled bacteria to lung tissue while maintaining a satisfactory signal to noise ratio in both channels. We defined the relative visibility as the expected spectral ratio value of SmartProbe labelled *P. aeruginosa* divided by the expected spectral ratio value of lung tissue. The relative visibility, seen in Fig. 2

, is maximised at ~650 nm. However the signal to noise ratio in the long channel for both SmartProbe labelled bacteria and lung tissue is poor. As a practical compromise in this proof of concept demonstration, and influenced by the availability of mirrors, a cut off of 605 nm was chosen where relative visibility reaches 75% of the maximum, but signal amplitude has not dropped excessively.

### 2.2 Image processing

The 8100 cores of the imaging fibre are arranged in a tessellated square formation [23]. This core structure, as with all fibre bundles used for endomicroscopy, dominates the eye during imaging which can distract the user; thus it is often removed during post-processing. However, the core pattern was advantageous during our image processing algorithm as we could treat the fluorescence signal transmitted down each core independently. Thus it enabled us to perform ratiometric imaging on a core-by-core basis. Additionally, targeting the individual cores within the pattern is of particular importance in the realm of bacterial imaging where the bacterial targets or clumps may be ~3 µm in diameter and occupy only one or two cores within the fibre field of view. The image processing described in the following subsections of this paper is multistage: a core detection algorithm (§ 2.2.1), pairing of the cores between wavelength channels (§ 2.2.2), and normalisation and interpolation to produce a final ratiometric image (§ 2.2.3). The core detection algorithm required a brightfield, broad spectrum image of the fibre in each wavelength channel in order to spatially map core locations. The brightfield image was first convolved with a Mexican hat transform, then cores were selected from the inter-core cladding and the core centres were defined, producing a set of core locations, core sizes and their intensities. Pairing of the cores between wavelength bands was achieved through calculating distances between all points in the two sets and selecting the minimum values. The production of the final image was carried out through a simple linear interpolation after spectral ratio calculation and resulted in visualisation of both intensity information and the spectral ratio. All image processing was carried out using Python and performed offline. In future we will improve the efficiency of our image processing in order to make spectral ratio imaging real-time.

#### 2.2.1 Detection of cores from brightfield image and normalised core intensity

**Fig. 3:**
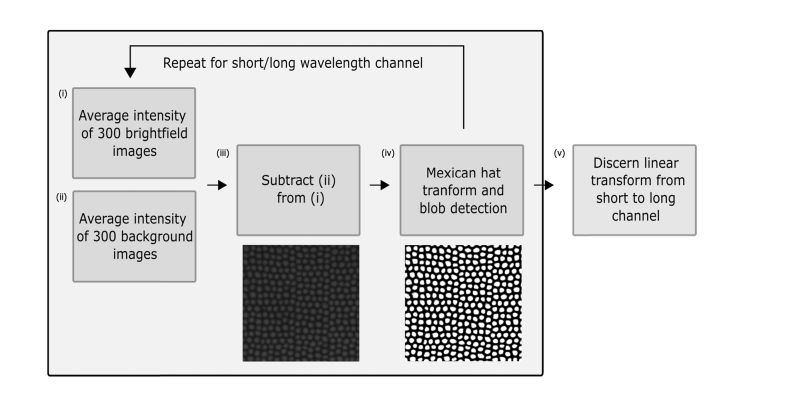
Initial, semi-supervised processing of data set that sets out parameters for core data extraction.

A series of brightfield images were taken to segment the cores and a series of background images were taken to enable subtraction of the autofluorescent fibre background. These images were taken immediately prior to each biological imaging session. A wide range of core detection algorithms exist, each with varying degrees of complexity and effectiveness [30] and in this work, we used a wavelet transform. Initially, 300 brightfield images and 300 background images were taken (Fig. 3 (i) and (ii)

) and these were averaged over intensity to produce single images of brightfield and background with low noise. The averaged background image was subtracted from the averaged brightfield image (Fig. 3 (iii)) and the resulting image was convolved with a Mexican hat wavelet function (Fig. 3 (iv)). Convolving the brightfield image with this transform effectively suppressed pixels in the inter-core regions and elevated the values of the core pixels themselves. This allowed for a simple blob detection algorithm to accurately detect and register the cores within the image with high accuracy.

Once the cores were detected, the centre of mass (COM) of the pixels within each core was calculated and this established what we refer to hereafter as the core location. Where the algorithm unavoidably fails is in cases of physical artefacts within the fibre resulting in very low light transmission at some cores. Cores such as these were either undetected entirely or, as a result of drastically varying intensity profile, were detected as multiple cores. In the latter case, these cores were automatically removed from the analysis at this stage. Cores which were not detected at all were ignored, and if detected in one channel but not the other were removed from the set.

**Fig. 4:**
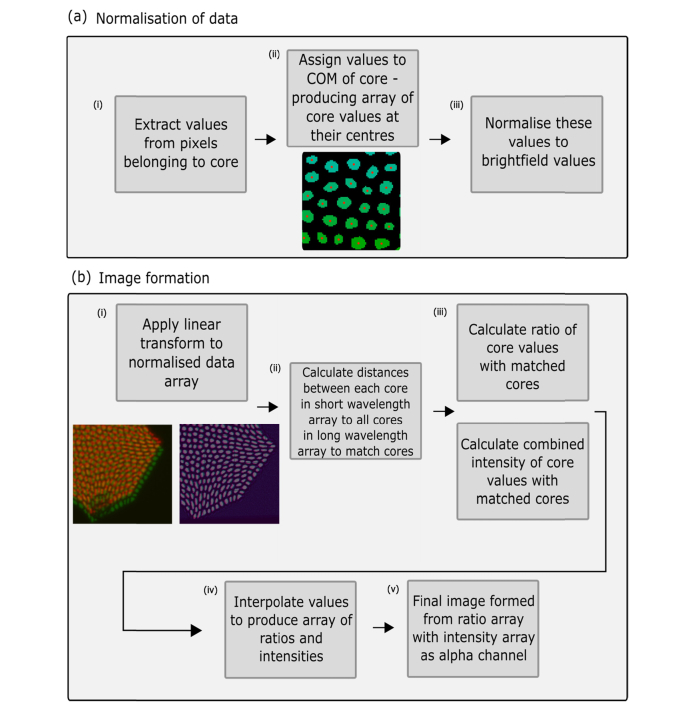
Schematic of processing methodology used to analyse data and produce final images. (a) How wavelength dependent propagation of light through different cores is accounted for through normalisation of the data with a brightfield image. (b) How spectral ratio values are calculated following a core matching protocol and interpolation.

The sum value of pixels within each core was calculated (Fig. 4 (a) (i)

) and assigned to its COM (Fig. 4 (a) (ii)). This step was carried out on both the brightfield images in each wavelength channel and the target images in each wavelength channel. This allowed for a normalisation step to take place to account for wavelength dependent propagation of each core (Fig. 4 (a) (iii)).

#### 2.2.2 Matching of cores between wavelength bands

As a result of a combination of chromatic aberrations due to the broad spectral range of imaging and a low tolerance of the method to minor system misalignment (each pixel spans approximately 0.3 µm^2^ of the fibre face), the alignment between wavelength channels was too poor for a simple core matching process to be effective. In order to ensure reliable matching of cores between each wavelength channel, our algorithm included a supervised alignment step that set out new core location coordinates according to a linear geometric transform from the fibre corner locations (Fig. 3 (v)). Subsequent to applying the transform (Fig. 4 (b) (i)), the cores were reliably matched by calculating the minimum distances between all cores in each channel (Fig. 4 (b) (ii)). Cores for which no match could be found were discarded from the data set. In any typical experiment this accounted for < 1% of the total number of cores and was due to cores that did not efficiently propagate light and were not detected in one channel or the other.

#### 2.2.3 Final image reconstruction

Once all cores were matched between channels, it was trivial to calculate their spectral ratio value (Fig. 4 (b) (iii)) according to (1) and map these onto a perceptually uniform colour scale. Throughout this paper we calculated the spectral ratio as short:long so that larger spectral ratio values represent a larger contribution of the short wavelength channel to the overall image. We linearly interpolated between cores to produce our final images (Fig. 4 (b) (iv)), but other interpolation types could be implemented if desired. By also using the original intensity values as an alpha channel (Fig. 4 (b) (iii)), final images were produced that display both ratiometric information and intensity information (Fig. 4 (b) (v)).

### 2.3 Experimental methods

Here we describe a highly controlled methodology using a 1951 USAF test target initially used to validate our imaging system and then describe the *ex vivo* lung model that we present as an exemplar of a potential clinical application.

#### 2.3.1 USAF test target characterisation

**Fig. 5:**
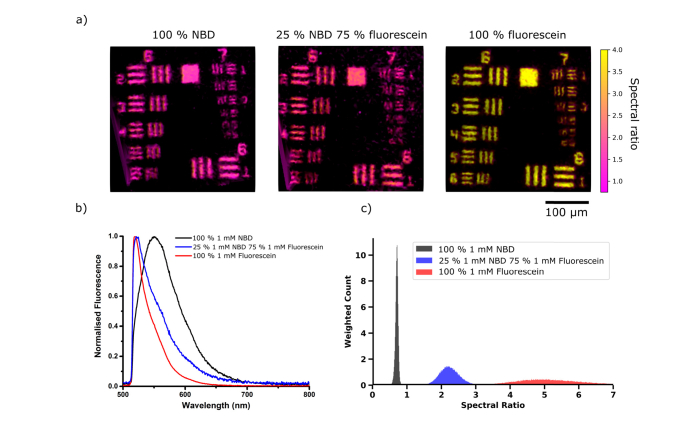
(a) Images from negative USAF target with (left to right) 100% 1 mM NBD, 25% 1 mM NBD 75% 1 mM fluorescein, and 100% 1 mM fluorescein with spectral ratio values clipped to 0.75 - 4. (b) Spectra of the three solutions taken at an illumination wavelength of 470 nm at 5.5 µW, using a commercial spectrometer (USB2000 + VIS-NIR-ES, Ocean Optics). (c) Histograms of the spectral ratio values contained within USAF images summed over the whole image for ten sequential frames. The histograms have been weighted by intensity and normalised by area for ease of comparison. Broadening of the 100% fluorescein histogram is due to a low signal to noise ratio above the cut-off wavelength at 605 nm. Note that a shift of the spectra towards shorter wavelengths corresponds to an increase in the spectral ratio calculated.

We confirmed that differences between overlapping fluorescent targets could be resolved with our system through a simple imaging experiment. Firstly, to acquire both structural and ratiometric resolution we imaged a negative USAF test target placed above varying mixtures of two fluorophores whose emission was known. We chose 1 mM NBD and 1 mM fluorescein as fluorophores with notably different spectral emission, see Fig. 5

.

#### 2.3.2 Ex vivo human lung model

Ethics statement: All experiments using human samples *ex vivo* were performed following approval of the appropriate regional ethics committee (REC), NHS Lothian (reference 16/LO/1883), and with consent.

**Fig. 6:**
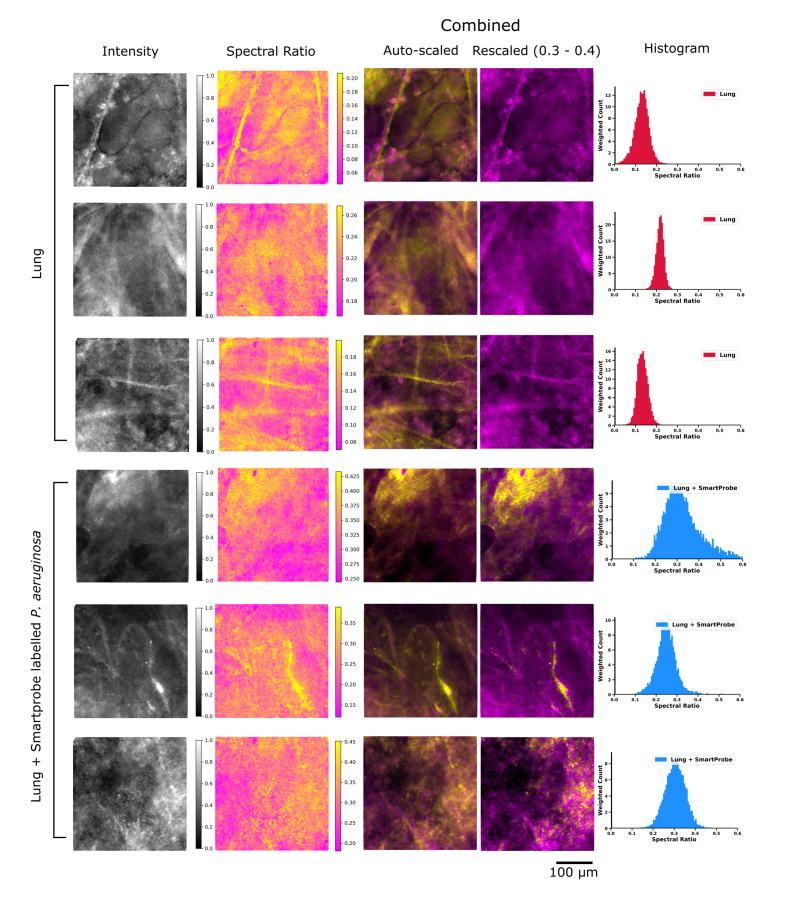
(top three rows) Typical examples of imaging lung tissue in an *ex vivo* lung perfusion (EVLP) model prior to instillation of labelled bacteria. (bottom three rows) Typical examples of EVLP lung imaging after *in situ* delivery of SmartProbe labelled *P. aeruginosa*. All images are the same FOV presented in four modes. (from left to right) Intensity image as would appear on a widefield fluorescence fibred OEM system; spectral ratio image (auto-scaled); combined image with auto-scaling; combined image with scaling clipped from a spectral ratio of 0.3 to 0.4 to enhance SmartProbe labelled *P. aeruginosa* visibility (see Visualization 2); histograms of the spectral ratio contained with the image. Selected images are shown enlarged in Fig. 7.

An *ex vivo* ventilated human lung model, as described in [8], was set up to test the system's capability of enhancing the contrast between lung tissue autofluorescence and pre-labelled bacteria. A whole human lung was ventilated and a bronchoscope inserted into the airways. The working channel of the bronchoscope was then used to pass Panoptes into the lung. The narrow diameter of the packaged fibre bundle (~1.4 mm) enabled access to the distal alveolar regions via a transbronchial approach. Baseline imaging of lung tissue was carried out, see Fig. 6

and Fig. 7

**Fig. 7:**
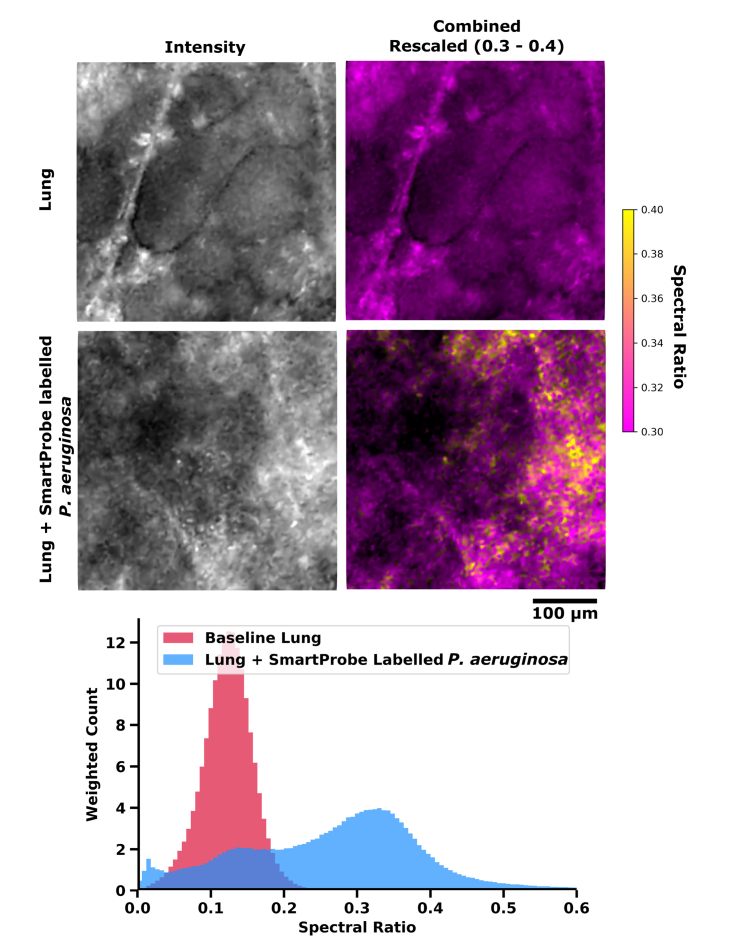
(Top) Typical *ex vivo* imaging of lung tissue from EVLP model in alveolar space before and after *in situ* instillation of pre-labelled bacteria. Greyscale images show how imaging appears in the widefield imaging system without spectral ratio analysis enabled. Coloured images display spectral ratio and intensity with rescaling of spectral ratio to 0.3 - 0.4 to enhance SmartProbe labelled *P. aeruginosa* visibility. (Bottom) Typical histogram of spectral ratio across 300 frame videos of lung before and after delivery of labelled bacteria. A tabulated series of images can be seen in Fig. 6.

, before *in situ* delivery via the capillary channel of Panoptes of SmartProbe labelled *P. aeruginosa* (100 µL, 1 × 10^8^ CFUml^−1^), similar to that described in detail elsewhere [20].

## 3. Results and discussion

### 3.1 USAF test target characterisation results

Optical resolution was limited by the core to core coupling [30] and core spacing in the imaging fibre (even though the visual core pattern was successfully removed). Resolution of lines in group 7 element 3 of the chart (the elements have line widths of 3.10 µm), see Fig. 5 (a), was in agreement with that expected from previous observations [23]. Meanwhile change in colour in the images (also shown as the histograms of spectral ratio in Fig. 5 (c)) demonstrated that subtle spectral differences between fluorescent targets could be visualised despite illuminating with a single wavelength band and without gathering full spectral information of the targets themselves. The validity of the ratiometric imaging result was confirmed by acquiring emission spectra using a commercial spectrometer in conjunction with imaging (Fig. 5 (b)).

### 3.2 Ex vivo human lung and SmartProbe pre-labelled Pseudomonas aeruginosa

Imaging was continuously performed in the field of view in the lung region where SmartProbe labelled *P.aeruginosa* was delivered and an increase in spectral ratio suggesting the presence of bacteria could be immediately visualised. We observed lung tissue structure as strands and other structures throughout the field of view. While it is apparent lung tissue without fluorophores has some variation in spectra (see top images of Fig. 6 and Visualization 1), the spectral ratio largely remains below a value of 0.3. Meanwhile, after the addition of fluorescently labelled bacteria we observed a higher spectral ratio. With a scale range chosen to highlight this contrast, yellow features in the regions where we delivered labelled bacteria became apparent. These were absent from the images of lung tissue plotted at this scale (Fig. 6 and enlarged in Fig. 7). See Visualization 2 for accompanying videos from Fig. 6.

Detection of features such as labelled bacteria in typical widefield fluorescent fibred OEM systems when spectra are very similar typically depends on recognising sets of characteristic behaviours. An example of this is in the interpretation of the bacterial burden within the distal lung following the delivery of molecularly targeted SmartProbes. In our recent experience, this was highlighted by punctate ‘twinkling’ within a video [20], believed to be a result of suspended bacteria in the FOV moving between individual cores of the fibre. In addition to this punctate signal (e.g. sixth row of Fig. 6) our images provide evidence that bacteria may adhere to the alveolar tissue itself (e.g fifth row of Fig. 6) when delivered locally, and consequently adherent bacteria may not appear as a twinkling and might not be noticed by looking for this characteristic. Crucially, this feature alone may underestimate the identification of bacteria within an OEM image. By combining spectral ratio imaging with knowledge of these behaviours, our system can improve the interpretation of fibre-based OEM.

Our approach of using an optical chopper to divide the optical path into short and long wavelength channels in order to calculate spectral ratio values is not the only way to achieve this outcome. By taking images in each spectral band in sequence to later recombine into a single image, we assume that the FOV remains unchanged between each image. Although each frame is captured with an exposure time of 50 ms, this assumption fails if the target moves rapidly, as happens during fibred imaging of ventilated lungs. We found that motion artefacts were apparent in many data sets and future iterations of the system may feature a separate camera for each optical channel. This would allow simultaneous image acquisition of the FOV and likely significantly improve motion artefacts within the system.

## 4. Conclusion

Widefield fibred OEM systems have the potential to be widely applicable, especially by multiplexing such a system with labels targeted to disease. When tissue autofluorescence contributes to a significant background, features within the image marked by exogenous fluorophores can become less significant or even indistinguishable. We have developed a simple ratiometric fibred fluorescence imaging system and demonstrated its application to the visualisation of fluorescently labelled bacteria in a whole human lung model. The images produced using this system show that contrast enhancement between spectrally broad and overlapping emission sources can be readily achieved in a cost-effective platform.

Coupling this simple system with a single use disposable packaged fibre [24] provides an entire platform with significant clinical utility. Future work will include assessing performance in lung tissue with existing infection, in exploiting the capability to unmix multiple targets over multiple wavelengths, and in a label-free imaging regime of lung and other tissues, see appendix, and Visualization 1.

## Figures and Tables

**Fig. 8:**
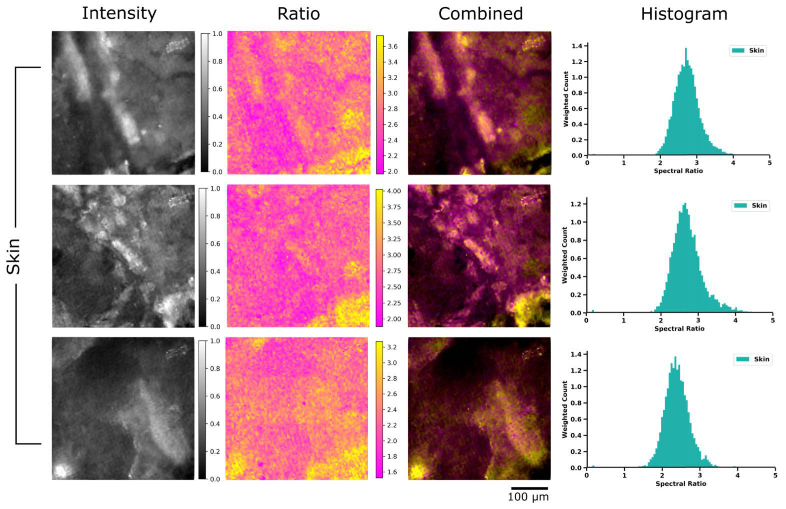
Typical examples of skin tissue. From left to right, intensity only image; spectral ratio only image with auto-contrast; combined image; histogram of the spectral ratio within the image weighted by intensity.

## Appendix

**Skin tissue**: As a demonstration of our system within label-free imaging contexts, skin tissue was imaged, see Fig. 8. Intensity mode represents the sample as it would appear in a widefield fluorescence imaging system showing how this is sufficient for clear visibility of tissue structure only. While these types of greyscale images are often false-coloured to give an impression of colour, spectral information pertaining to the structure remains unknown. With our spectral ratio images, spectrally different features are given a colour contrast and we can see that this spectral ratio does not typically follow intensity information. Once the two images are combined, we can attribute different structural features with a range of spectral ratios.
